# A pediatric case of productive cough caused by novel variants in *DNAH9*

**DOI:** 10.1038/s41439-020-00134-6

**Published:** 2021-01-15

**Authors:** Kazuhiko Takeuchi, Yifei Xu, Satoru Ogawa, Makoto Ikejiri, Kaname Nakatani, Shimpei Gotoh, Satoko Usui, Sawako Masuda, Mizuho Nagao, Takao Fujisawa

**Affiliations:** 1 Department of Otorhinolaryngology, Head & Neck Surgery, Mie University Graduate School of Medicine, Tsu, Mie Japan; 2 Electron Microscopy Research Center, Mie University Graduate School of Medicine, Tsu, Mie Japan; 3 Department of Central Laboratories, Mie University Hospital, Tsu, Mie Japan; 4 Department of Genomic Medicine, Mie University Hospital, Tsu, Mie Japan; 5 Department of Drug Discovery for Lung Diseases, Graduate School of Medicine, Kyoto University, Kyoto, Japan; 6 Department of Otorhinolaryngology, National Hospital Organization Mie National Hospital, Tsu, Mie Japan; 7 Institute for Clinical Research, National Hospital Organization Mie National Hospital, Tsu, Mie Japan

**Keywords:** Respiratory tract diseases, Medical genomics

## Abstract

We report the first Japanese case of primary ciliary dyskinesia caused by *DNAH9* variations. The patient, a 5-year-old girl, had repeated episodes of productive cough after contracting the common cold at the age of 1 year and 6 months. She did not have a situs abnormality or congenital heart defect. We identified two novel *DNAH9* variants, NM_001372.3: c. [1298C>G];[5547_5550delTGAC], (p.[Ser433Cys];[Asp1850fs]).

Primary ciliary dyskinesia (PCD) is a rare genetic disorder that causes impaired ciliary function and occurs in approximately 1 in 20,000 live births^1^. PCD is inherited in an autosomal recessive or X-linked manner. The symptoms of PCD are diverse, including situs inversus, chronic oto-rhino-pulmonary infections, and infertility, and they can vary among patients. This heterogeneity makes the diagnosis of PCD challenging, particularly when situs inversus is absent and the other symptoms are mild. Patients with PCD have a high prevalence of persistent wet cough throughout life^2^. As a result, patients with PCD are sometimes misdiagnosed and treated for asthma^3^. Herein, we report a pediatric case of PCD in which the patient had a productive cough and had been treated for asthma.

A 5-year-old girl was referred to our hospital because of a recurrent productive cough. She was born at term and did not experience chest symptoms in the neonatal period. She did not have a situs abnormality or congenital heart defect. The first year of life was uneventful. From the age of 1 year and 6 months, she had repeated episodes of productive cough after contracting the common cold. She was diagnosed with asthma by her family doctor and administered procaterol (Meptin^®^ tablets) and inhaled budesonide (Pulmicort^®^ Turbuhaler^®^), but they were not effective.

Her eardrums were normal bilaterally (Fig. 1a). Nose X-ray showed opacification in her right maxillary sinus, suggesting sinusitis (Fig. 1b), but her chest X-ray was normal (Fig. 1c). Serum total IgE was 3 IU/mL, and no specific IgEs were detected. At the age of 5 years, her nasal nitric oxide (NO) concentration was extremely low (44 ppb), as measured with a chemiluminescence analyzer (CLD 88SP; ECO PHYSICS AG, Duernten, Switzerland). Nasal NO production, calculated by multiplying the nasal NO concentration (ppb) by the sampling flow rate (0.33 L/min), was 14.5 nL/min, which was lower than the PCD-specific NO cutoff value of 77 nL/min (sensitivity, 0.98; specificity, 0.999)^4^.

**Fig. 1:**
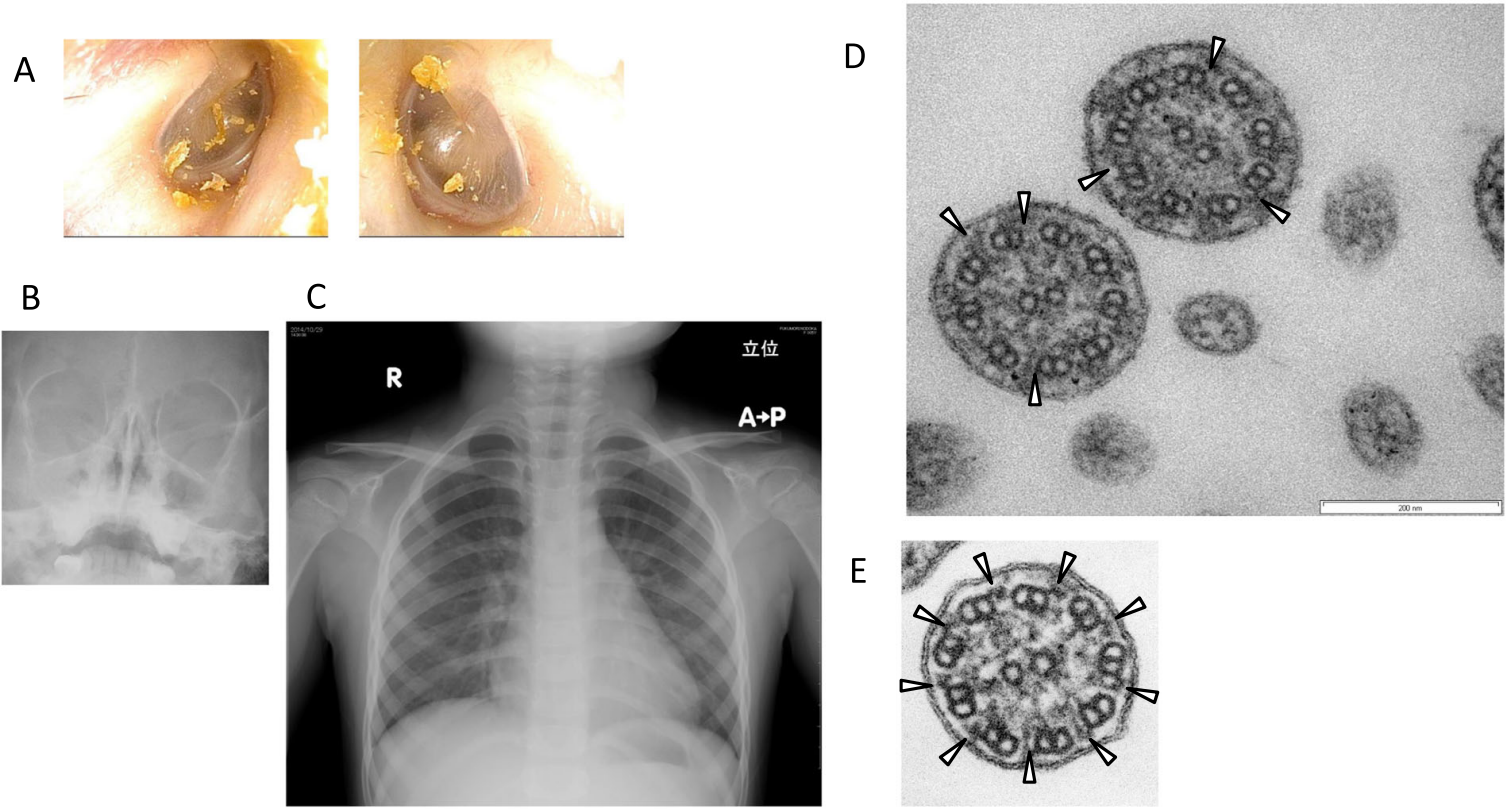
Clinical images and electron microscopy. Her eardrums were normal **a**. Nose X-ray showed opacification in her right maxillary sinus **b**. Her chest X-ray was normal **c**. Outer dynein arms (arrows) were not clearly seen in electron microscopy of a biopsy specimen from her nasal mucosa **d**. Outer dynein arms (arrows) are seen in a cilium of a normal subject **e**.

PCD was suspected, and further examinations were performed. In electron microscopy (JEM-1011; JEOL, Tokyo, Japan) of a biopsy specimen from the nasal mucosa, the outer dynein arms were not seen clearly (Fig. 1d, e). Whole-exome sequencing^5^ revealed two heterozygous mutations in dynein axonemal heavy chain 9 (DNAH9; NM_001372.3: c.[1298 C > G];[5547_5550delTGAC], p.[Ser433Cys];[Asp1850fs]), neither of which has been reported previously. Ser433 sits within dynein heavy chain domain 1 of DNAH9. This domain interacts with other heavy chains to form dimers and interacts with intermediate chain-light chain complexes to form a basal cargo binding unit. Asp1850 is located upstream of the hydrolytic ATP-binding dynein motor region D1, and this frameshift variant is predicted to eliminate the downstream dynein motor region. Finally, these two variants were validated via PCR and Sanger sequencing with a 3500 Series Genetic Analyzer (Life Technologies, Carlsbad, CA, USA)^5^. Sanger sequencing confirmed the compound heterozygous mutations in DNAH9 identified by whole-exome analysis in the proband (Fig. 2). The patient’s father carried only the latter mutation (Fig. 2), and her mother carried only the former mutation (Fig. 2); these findings confirmed that each mutation was inherited from a different parent.

**Fig. 2:**
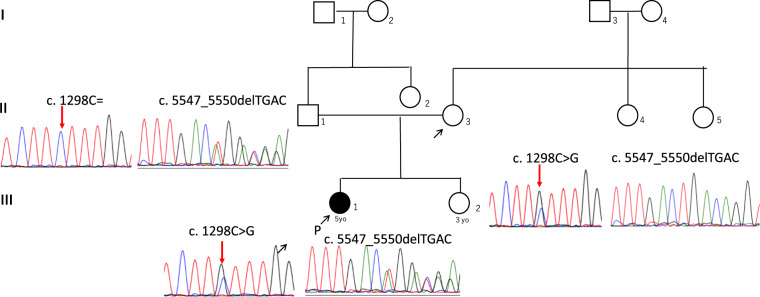
Family pedigree with the results of Sanger sequencing. Compound heterozygous mutations in *DNAH9* are shown.

This study was approved by the Ethics Committee of Mie University Graduate School of Medicine (approval number 1363), and written informed consent was obtained from this patient and her parent.

PCD is a genetically heterogeneous disorder; pathogenic mutations in 44 genes have been reported to cause PCD^6^. In Japan, the genes most commonly causing PCD are dynein regulatory complex subunit 1 (49%), *DNAH5* (31%), and *DNAH11* (12%)^7^. *DNAH9* was recently reported as a PCD-causing gene^8,9^ and encodes the heavy chain subunit of axonemal dynein, a large multisubunit molecular motor^8^. Axonemal dynein attaches to microtubules and hydrolyzes ATP to mediate the movement of cilia. DNAH9 and its partner DNAH5 localize to the type 2 outer dynein arms of the distal cilium^9^. This is the first case of *DNAH9* mutations with *situs solitus* and the first case in Japan.

Loges et al.^8^ reported loss-of-function mutations in *DNAH9* in 5 independent families causing situs abnormalities associated with subtle respiratory ciliary dysfunction. Consistent with the observed subtle respiratory phenotype, high-speed video microscopy has demonstrated distally impaired ciliary bending in *DNAH9* mutant respiratory cilia^8^. *DNAH9* mutations reduce cilia function, but some respiratory mucociliary clearance may be retained^9^. Thus, unlike typical cases of PCD, cases with *DNAH9* mutations are reported to have subtle respiratory signs and symptoms^8,9^.

PICADAR is a simple clinical diagnostic tool for PCD that has good accuracy and validity^2^. In addition to persistent wet cough that started in early childhood, our patient had only term birth and sinusitis as characteristic features of PCD. She did not have chest symptoms in the neonatal period, situs inversus, or a congenital heart defect. Thus, her PICADAR score was only 3, which corresponds to a 1.9% likelihood of a diagnosis of PCD^2^.

On electron microscopy images of tissue from the patient, the outer dynein arms were not clearly visible. Fassad et al.^9^ reported that outer dynein arm defects affect 35–89% of axonemes in individuals carrying *DNAH9* mutations, with significant numbers of outer dynein arms in most cases remaining undisturbed. Because we did not examine many cilia in this case, it is impossible to calculate the percentage of axonemes affected.

Recently, it was reported that *DNAH9* polymorphisms are associated with asthma and bronchial hyperresponsiveness in response to early life exposure to tobacco smoke^10^. The causal relationship between *DNAH9* polymorphisms and bronchial hyperresponsiveness is not known, but the partial ciliary dysfunction caused by *DNAH9* mutation may reduce the efficiency of respiratory clearance mechanisms, thus promoting microbial colonization and inflammation, leading to bronchial hyperresponsiveness.

In summary, compound heterozygous mutations in *DNAH9* were found in a pediatric patient with persistent productive cough. Mutations in this gene might play a role in cough in pediatric patients.

## Data Availability

The relevant data from this Data Report are hosted at the Human Genome Variation Database at 10.6084/m9.figshare.hgv.2957 and 10.6084/m9.figshare.hgv.2960.
